# Exterge Oculum

**Published:** 1883-09

**Authors:** C. R. Eggemann

**Affiliations:** Surgeon to the City Eye and Ear Clinic of Detroit, Mich.; 170 Randolph Street


### ARTICLE IV.

EXTERGE OCULUM. By C. R. EGGEMANN, M.D., Surgeon to the City Eye and Ear Clinic of Detroit, Mich.

As there is no literature on this subject, I will proceed to give the history of it. Dr. Liebold, of New York, uses this method with very few exceptions, which will be spoken of later on. Patients who had enucleations performed complained that the artificial eye had not sufficient motion, as there was not stump enough to give the requisite amount of motion expected by them. This induced Dr. Liebold to use this method, and I look upon him as the inventor of this operation, he having taught and practiced it for many years. The indications for the operation I will give in the Doctor's own words: "I regard all those for good, in which enucleation is practiced, except in cases of intraocular tumors, or where a tumor has invaded or is liable to invade the sclera."

The steps to the operation are as follows: The patient being anæsthetized, the lids are held apart by a stop speculum, and the bulbus firmly with the fixation forceps, then the eye is transfixed with a Graafe knife in the horizontal diameter, about two lines from the sclero-corneal junction, or posterior to the ciliary body, then cutting either upwards or downwards as the operator thinks best; the fixation forceps are removed after completing the incision, the flap is grasped by the same, and the remaining portion is cut away with scissors curved on the flat. The principal point is to remove all the ciliary nerves from their entrance into the carpus ciliare. Then interior of the eye is *wiped out*, as it were, with small pellets of lint, the latter being held with the fixation forceps, removing thereby the retina, choroid and ciliary nerves, leaving nothing but the white sclerotic with the muscles attached

The conjunctiva can be united with two sutures over the opposite recti muscles, or the sutures can be omitted and the sclera allowed to become glued together by the proliferation of cell elements. The after treatment consists in cold water dressings, or the application of an icebag. Apply as long as patient feels comfortable with it, being guided by the feelings of the patient. There is no trouble from secondary hæmorrhage as it sometimes happens after an enucleation, in cases where the arteries are diseased, for in the former the arteries entering the eye are twisted by the motion of wiping out the eye, while in the latter they are cut transversely. The swelling is inconsiderable and subsides soon. The stump is larger and moves better, as all the external muscles remain in their original position. The objection that an artificial eye will irritate easier is groundless, if it is too large it will irritate the branches from the nervus naso-ciliaris, whether the sclera is preserved or not.

What spurred me on to write this article, is the limited experience I have had with this operation. Jno. K., æt. 37, laborer in the car shops of Detroit, was hit in the right eye with a piece of wood, July 24, 1882. He came to my office a week later to consult me. Status præsens, right eye, chemosed conjunctiva protruding from external canthus, cornea had been ruptured in the whole horizontal diameter, and became agglutinated again, iris adherent to inner surface corresponding to the laceration of the cornea. Tension. Complains of terrific pain in and around eye. Left eye shows signs of sympathetic irritation, conjunctiva considerably congested, profuse lachrymation, iris contracted, but responds well, also photophobia present. Advised enucleation, explaining to him the danger he was in of losing the left, his sound eye, to which he willingly consented. Assisted by Mr. Sommers, then a medical student, I proceeded to operate. Ether was given to him to blunt the sensation, I applied the speculum, and had completed the circular cut through the conjunctiva around the cornea previous to detaching the muscles, when an accident occurred, caused by the patient holding his breath, which opened slightly the old wound, allowing the contents to partially escape. Knowing that I could not use the strabismus hook to lift up the muscles without having the eye collapse, the teachings of Dr.

Liebold came to my rescue. Instead of enucleating as I intended, I *wiped* the contents of the eye out as above described. I did not apply any sutures. The after treatment consisted in cold water dressings. Fitted the patient with an artificial eye two weeks after the operation. The movements are good in, out, up or downwards. I have seen the patient several times since, and he is well pleased saying that he has motion to the artificial eye, while a fellow workman, who had an enucleation performed, has not any. So much for an accident. Hoping this operation will in time supersede enucleation and optico, ciliary neurectomy, or any of the operations for total staphyloma of the cornea, I will close with a few remarks on the title of this subject. Dr. Liebold has named the operation "Evacuatio Bulbi." We have the contents of the eye escaping in the operations for total staphyloma of the cornea, and I think this title does not apply to the operation as well as the one I have selected, namely, "Exterge Oculum," which means, *wipe out the eye.*

170 RANDOLPH STREET.

---

